# Tebuconazole Fungicide Induces Lipid Accumulation and Oxidative Stress in HepG2 Cells

**DOI:** 10.3390/foods10102242

**Published:** 2021-09-22

**Authors:** Hyuk-Cheol Kwon, Do-Hyun Kim, Chang-Hee Jeong, Yea-Ji Kim, Jong-Hyun Han, Su-Jin Lim, Dong-Min Shin, Dong-Wook Kim, Sung-Gu Han

**Affiliations:** 1 Department of Food Science and Biotechnology of Animal Resources, Konkuk University, Seoul 05029, Korea; rnjs1024@konkuk.ac.kr (H.-C.K.); secret311@konkuk.ac.kr (D.-H.K.); dpwl961113@konkuk.ac.kr (Y.-J.K.); hyeon4970@konkuk.ac.kr (J.-H.H.); tnwlsdl1110@konkuk.ac.kr (S.-J.L.); s900704@konkuk.ac.kr (D.-M.S.); 2 Microbiology and Functionality Research Group, World Institute of Kimchi, Gwangju 61755, Korea; jeongch@wikim.re.kr; 3 Department of Poultry Science, Korea National College of Agriculture and Fisheries, Jeonju 54874, Korea; poultry98@korea.kr

**Keywords:** tebuconazole, lipid accumulation, lipid metabolism, oxidative stress, non-alcoholic fatty liver disease

## Abstract

Tebuconazole (TEB), a triazole fungicide, is frequently applied to agriculture for the increase of food production. Although TEB causes liver toxicity, its effects on cellular lipid accumulation are rarely investigated. Therefore, this study aimed to study the effects of TEB on lipid metabolism and accumulation in HepG2 cells. HepG2 cells were exposed to 0–320 µM TEB for 1–24 h. TEB (20–80 µM, 24 h)-treated cells showed lipid accumulation. Further, TEB (20–80 µM, 1–12 h) increased the nuclear translocation of peroxisome proliferator-activated receptors and the expression of lipid uptake and oxidation-related markers such as cluster of differentiation 36, fatty acid transport protein (FATP) 2, FATP5, and carnitine palmitoyltransferase 1. Oxidative stress levels in TEB-treated cells (20–80 µM, 24 h) were higher, compared to those in the control. TEB (20–80 µM, 24 h) also induced the loss of mitochondrial membrane potential and lower levels of microsomal triglyceride transfer protein in the cells. Thus, TEB can induce lipid accumulation by altering the expression of lipid-metabolizing molecules and can therefore impair lipid metabolism. Our data suggest that human exposure to TEB may be a risk factor for non-alcoholic fatty liver disease.

## 1. Introduction

Tebuconazole (TEB), a triazole fungicide, is often applied to agriculture for the increases of crop yield and food production [1]. TEB acts by interfering with fungal ergosterol biosynthesis through the inhibition of cytochrome P450 (CYP) 51 (lanosterol 14 alpha-demethylase), an essential component of fungal cell membrane integrity [2]. However, extensive use of TEB to control a broad spectrum of fungal diseases causes the accumulation of pesticide residues in food, water, and soil, thereby resulting in various harmful effects on human health [3]. Indeed, previous studies have reported that TEB residues were detected in stream and surface water at concentrations of up to 175–200 µg/L and that the risk value for the consumer chronically exposed to TEB residues in agricultural and animal origin commodities had reached up to 9.65 mg/kg [4,5]. Considering that both the acceptable daily intake and acute reference dose for TEB are 0.03 mg/kg body weight, and that human exposure to TEB residues may be frequent, TEB has the potential to cause toxicity in the human body, including hepatotoxicity [4].

Ingested TEB residues are absorbed in the gastrointestinal tract and are mainly metabolized to TEB-1-hydroxy and TEB-carboxylic acid via CYP-mediated oxidation in the liver for excretion into urine and feces [6]. However, CYP catalytic cycles for xenobiotic biotransformation (e.g., TEB) generate various reactive oxygen species (ROS), including hydrogen peroxide and superoxide anion [7]. Such ROS generation causes a redox imbalance and oxidative stress in the liver [7]. According to a previous study, excessive oxidative stress led to the peroxidation of lipids such as cholesterol and polyunsaturated fatty acids, resulting in the development and progression of non-alcoholic fatty liver disease (NAFLD) [8]. The liver is well known to be an essential organ for lipid metabolism and lipid homeostasis, and is involved in the synthesis, export, and redistribution of fatty acids [9]. Further, the hepatic lipid metabolism comprises four major pathways, including fatty acid uptake, *de novo* lipogenesis, oxidation of fatty acids, and transport of lipids in very low-density lipoproteins (VLDL) [10]. However, an imbalance of hepatic lipid homeostasis has induced lipid accumulation in the liver and subsequently caused pathogenic conditions, such as NAFLD [10].

NAFLD is defined as fat accumulation of no less than 5% in the liver without secondary factors, such as alcohol or drugs [11]. The prevalence of NAFLD is approximately 25% worldwide and varies by region: 13% in Africa, 23% in Europe, 27% in Asia, 32% in the Middle East, and 55% in the Americas [12]. The economic and societal burden of NAFLD has been assessed to be $292 billion dollars in the USA [13]. However, only a few pharmaceutical therapies are available for treating NAFLD [14]. Recently, a mechanistic study on the pathogenesis of NAFLD has been conducted extensively for its treatment and prevention [15]. In a recent mechanism study on NAFLD pathogenesis, a multiple-hit hypothesis has received much attention [16]. In particular, lipid accumulation, lipid metabolism, and oxidative stress are known as important factors in the multiple-hit hypothesis on NAFLD development and progression [16].

Taken together, despite growing concerns regarding the adverse effects of TEB residues in drinking water and food on human health, the effects of TEB on hepatic lipid accumulation and lipid metabolism have not been entirely elucidated. Thus, this study aimed to examine the effect of TEB on NAFLD development and progression, particularly lipid accumulation and lipid metabolism-related mechanisms in HepG2 cells.

## 2. Materials and Methods

### 2.1. Chemicals and Reagents

TEB was purchased from Sigma-Aldrich (St. Louis, MO, USA). Dulbecco’s modified Eagle’s medium, fetal bovine serum, penicillin/streptomycin, and 0.05% trypsin and 0.53 mM ethylenediaminetetraacetic acid solution were obtained from Welgene Inc. (Gyeongsan, Daegu, Korea). Phosphate-buffered saline (PBS) was purchased from Lonza (Walkersville, MD, USA). N-acetylcysteine (NAC) and dimethyl sulfoxide (DMSO) were obtained from Amresco (Solon, OH, USA). Lipofermata was purchased by Cayman Chemical Co. (Ann Arbor, MI, USA). The Pierce bicinchoninic acid protein assay kit was provided by Sigma-Aldrich (St. Louis, MO, USA). Nitrocellulose membranes and enhanced chemiluminescence reagents were supplied by GE Healthcare Bio-Sciences (Pittsburgh, PA, USA) and Thermo Fisher Scientific (Pittsburgh, PA, USA), respectively.

### 2.2. Cell Culture and Treatments

Human liver HepG2 cells (America Type Culture Collection, Rockville, MD, USA) were maintained in Dulbecco’s modified Eagle’s medium supplemented with 10% fetal bovine serum and 1% penicillin/streptomycin in CO_2_ incubator containing 5% CO_2_ at 37 °C. The culture medium was changed every 2 days, and the cells were sub-cultured into 96-well plates, 6-well plates, and T-25 flasks (SPL Life Sciences Co., Ltd., Pocheon, Korea) when they reached 80–90% confluency using 0.05% trypsin and 0.53 mM ethylenediaminetetraacetic acid. For the experiment, TEB was dissolved in DMSO at a concentration of 40 mM. The cells were treated with 0–320 µM TEB or vehicle (DMSO) control for 1–24 h. The concentration range of TEB for cell treatment was chosen from previous studies [17,18].

### 2.3. Cell Viability Assay

An MTT assay was conducted to evaluate cell viability using 3-(4,5-dimethylthiazol-2yl)-2,5-diphenyl-2H-tetrazolium bromide (MTT; Amresco, Solon, OH, USA), as described previously [19]. HepG2 were cultured into a 96-well plate and exposed to various concentrations of TEB (10, 20, 40, 80, 160, and 320 µM) or DMSO for 24 h (*n* = 3 wells/group). The media were replaced with 100 µL of fresh media and were added with 10 µL MTT solution (5 mg/mL in PBS). Subsequently, the cells were incubated in a CO_2_ incubator containing 5% CO_2_ at 37 °C for 3 h. After discarding 90 µL MTT solution, 180 µL acidic isopropanol was added into each well, and the plate was placed at 37 °C for 1 h. The optical density (OD) was measured at 570 nm and 630 nm using an Epoch spectrophotometer (BioTek Instruments, Winooski, VT, USA), and the percentage of cell viability was calculated with the following Formula (1):

Cell viability (%) = (OD _sample_/OD _control_) × 100 (1)

### 2.4. Lactate Dehydrogenase (LDH) Activity Assay

An LDH release assay was conducted to evaluate the cell membrane integrity using the Cytox 96^®^ non-radioactive cytotoxicity assay kit (Promega, Madison, WI, USA). HepG2 cells were cultured in a 96-well plate and exposed to various concentrations of TEB (10, 20, 40, 80, 160, and 320 µM) or DMSO for 24 h (*n* = 3 wells/group). To measure the maximum LDH release, 10× lysis solution was supplemented to the wells about 45 min before the end of the TEB treatment. Then, 50 µL of supernatants were transferred into a new 96-well plate. The 96-well plate was incubated at 25 °C for 30 min after adding the CytoTox 96^®^ reagent of 50 µL to each well. The 50 µL of stop solution was then added to each well. The OD was measured at 490 nm using a spectrophotometer, and the percentage of LDH release was calculated with the following Formula (2):

LDH release (%) = (OD _Experimental_ LDH release)/(OD _Maximum_ LDH release) × 100 (2)

### 2.5. Oil Red O Staining

Intracellular triglycerides and cholesterol esters were determined using Oil Red O (Sigma-Aldrich, St. Louis, MO, USA). HepG2 cells were grown in 6-well plates and treated with 20, 40, and 80 µM TEB or DMSO for 24 h with or without NAC pretreatment (5 mM in PBS) for 1 h (*n* = 3 wells/group). The cells were fixed with 10% formalin at 25 °C for 1 h, and then rinsed with 60% isopropanol. Oil Red O staining solution (1 mL) was added to each well. After incubation for 10 min, the cells were washed thrice with deionized distilled water for the removal of the unbound dye. The stained areas were imaged and captured using a Nikon Eclipse Ti2-U and Nikon Eclipse Ts2R camera, respectively (Nikon Co. Ltd., Tokyo, Japan). The quantified levels of captured images were assessed using ImageJ software version 1.52a (National Institutes of Health, Bethesda, MD, USA).

### 2.6. Determination of Nuclear Translocation of Peroxisome Proliferator-Activated Receptor (PPAR) Using Nuclear Fractionation

Protein levels of nuclear PPARγ and α were evaluated by nuclear fractionation. HepG2 cells were grown in 6-well plates and then treated with 20, 40, and 80 µM TEB or DMSO for 1 and 12 h (*n* = 3 wells/group). The cells were washed thrice with PBS and lysed using a hypotonic buffer containing 20 mM Tris (pH 7.4), 10 mM sodium chloride, 3 mM magnesium chloride, and protease inhibitor cocktail (Abbkine, Wuhan, China). The cell lysates were centrifuged at 600× *g* at 4 °C for 10 min after adding Triton X-100 to the cell lysates for the extraction of cytosolic proteins. The nuclear proteins were extracted using a cell extraction buffer containing 100 mM Tris (pH 7.4), 1 mM ethylenediaminetetraacetic acid, 2 mM sodium orthovanadate, 100 mM sodium chloride, 1 mM ethylene glycol-bis(β-aminoethyl ether)-N,N, N′N′-tetra acetic acid, 10% glycerol, 10% sodium dodecyl sulfate, 1 mM sodium fluoride, 0.5% sodium deoxycholate, 1% Triton X-100, 20 mM sodium pyrophosphate, 1 mM phenylmethylsulfonyl fluoride, 1 mM aprotinin, and 1 mM leupeptin. After resuspending the pellets using the cell extraction buffer, the samples were centrifuged at 14,000× *g* at 4 °C for 30 min. The protein concentrations were then analyzed using a bicinchoninic acid protein assay kit, and the samples were used for Western blotting.

### 2.7. Determining Protein Expression Using Western Blotting

Protein levels of the lipid metabolism associated cell signaling pathways were measured using Western blotting, as described previously [20]. HepG2 cells were grown in 6-well plates, and then exposed to 20, 40, and 80 µM TEB or DMSO for 12 and 24 h with or without NAC pretreatment (5 mM in PBS) for 1 h (*n* = 3 wells/group). The cells were lysed using a radioimmunoprecipitation assay buffer (Elpis Biotech, Daejeon, Korea). The cell lysates were then centrifuged at 18,000× *g* at 4 °C for 20 min. The protein concentrations were analyzed using a bicinchoninic acid protein assay kit. The protein samples (20 µg) were separated using sodium dodecyl sulfate-polyacrylamide gel electrophoresis. The -separated proteins were transferred onto a nitrocellulose membrane. After that, 3% nonfat milk buffer was used for blocking the membrane for 1.5 h at 25 °C. The membrane was incubated with the primary antibodies: anti-PPARα (1:1000 dilution; Santa Cruz, CA, USA), anti-PPARγ (1:3000 dilution; Cell Signaling Technology, Beverly, MA, USA), anti-cluster of differentiation 36 (CD36) (1:3000 dilution; Cell Signaling Technology, Beverly, MA, USA), anti-fatty acid transporter protein (FATP) 2 (1:3000 dilution; Abcam, Cambridge, MA, USA), anti-microsomal triglyceride transfer protein (MTTP) (1:3000 dilution; Abcam, Cambridge, MA, USA), anti-glyceraldehyde 3-phosphate dehydrogenase (GAPDH) (1:20,000 dilution; Merck Millipore, Darmstadt, Germany), and anti-Lamin B (1:5,000 dilution; Santa Cruz, CA, USA) at 4 °C from overnight to 3 days. After washing thrice with Tris-buffered saline with Tween 20, secondary antibodies (i.e., goat anti-rabbit IgG (1:5000 dilution; Enzo Life Sciences, Lausen, Switzerland) and donkey anti-goat IgG (1:5000 dilution; Santa Cruz, CA, USA) conjugated with horseradish peroxidase) were incubated for 1.5 h at 25 °C. Subsequently, the membrane visualized using enhanced chemiluminescence detection reagents. The quantified levels of protein expression were assessed using ImageJ software version 1.52a (National Institutes of Health, Bethesda, MD, USA).

### 2.8. Determining mRNA Expression Using Real-Time Polymerase Chain Reaction (RT-PCR)

Gene levels related to lipid metabolism were evaluated using RT-PCR. HepG2 cells were grown in 6-well plates and exposed to 20, 40, and 80 µM TEB or DMSO for 3–24 h with or without pretreatment with Lipofermata (20 µM in DMSO) and NAC (5 mM in PBS) for 1 h (*n* = 3 wells/group). RNA samples from the cells were extracted using TRIzol reagent (Ambion, Austin, TX, USA), and total RNA (2 µg) was reverse transcribed using the TOPscript RT DryMIX kit (Enzynomics, Daejeon, Korea) to synthesize cDNA. Gene expression levels were analyzed using an RT-PCR system (Roche LightCycler 96 System, Basel, Switzerland) and 2× RT-PCR Smart mix (BIOFACT CO., Ltd., Daejeon, Korea). The thermal cycling conditions were as follows: 95 °C, 15 min and then 60 cycles of denaturation (95 °C, 10 s), annealing (60 °C, 10 s), and extension (72 °C, 10 s). The 2^−ΔΔCt^ method was used to quantify gene expression levels. GAPDH gene expression levels were used as a control for normalization. The primers (BIONICS Co., Ltd., Seoul, Korea) were designed using the AmplifX software version 1.7.0 (Nicolas Jullien, CNRS, Aix-Marseille University, http://crn2m.univ-mrs.fr/pub/amplifx, accessed on 13 April 2021) (Table 1).

### 2.9. Cellular Oxidative Stress Measurement

Intracellular hydrogen peroxide and superoxide anion levels were evaluated using 2′,7′-dichlorofluorescin diacetate (DCFDA; Sigma-Aldrich, St. Louis, MO, USA) and dihydroethidium (DHE; Sigma-Aldrich, St. Louis, MO, USA). Briefly, HepG2 cells were cultured in 6-well plates and exposed to 20, 40, and 80 µM TEB or DMSO for 24 h with or without pretreatment with Lipofermata (20 µM in DMSO) for 1 h (*n* = 3 wells/group). After treatment with 10 µM DCFDA and DHE, the cells were incubated at 37 °C for 30 min in a 5% CO_2_ incubator. After washing thrice with PBS, green fluorescence (DCFDA-positive) and red fluorescence (DHE-positive) areas were imaged using a Nikon Eclipse Ti2-U and Nikon Elipse Ts2R camera. Fluorescence intensities were quantified using ImageJ software version 1.52a (National Institutes of Health, Bethesda, MD, USA).

### 2.10. Mitochondrial Membrane Potential (MMP) Measurement

MMP (ΔΨm) was estimated using the JC-10 mitochondrial membrane potential assay kit (Abcam, Cambridge, MA, USA), according to the instruction of the manufacturer. HepG2 were grown in Corning^®^ 96 well black polystyrene microplates (Sigma-Aldrich, St. Louis, MO, USA) and exposed to 20, 40, and 80 µM TEB or DMSO for 24 h with or without pretreatment with Lipofermata (20 µM in DMSO) (*n* = 3 wells/group). The JC-10 dye-loading solution of 50 µL was used to stain the cells, with incubation for 45 min in the dark. Assay buffer B (50 µL) was then added to each well, and the integrity of the MMP was evaluated as the ratio of the fluorescence of JC-10 aggregates (Ex/Em = 490/525 nm) and monomeric JC-10 (Ex/Em = 540/590 nm). The intensity of fluorescence was analyzed using a SpectraMax Gemini EM microplate reader (Molecular Devices, CA, USA), and the percentage of MMP loss was calculated as follows (3):

Loss of MMP (%) = (Fluorescence _525 nm_/Fluorescence _590 nm_) × 100 (3)

### 2.11. Data Analyses

Data are presented as mean ± standard error of the mean. The data were analyzed using SPSS-PASW statistics software version 18.0 for Windows (SPSS, Chicago, IL, USA). The significance was analyzed using a one-way ANOVA with post hoc Dunnett’s test and Student’s *t*-test. Statistical significance was defined at *p* < 0.05.

## 3. Results

### 3.1. Effects of TEB on Cell Viability and Damage in HepG2 Cells

**Figure 1:**
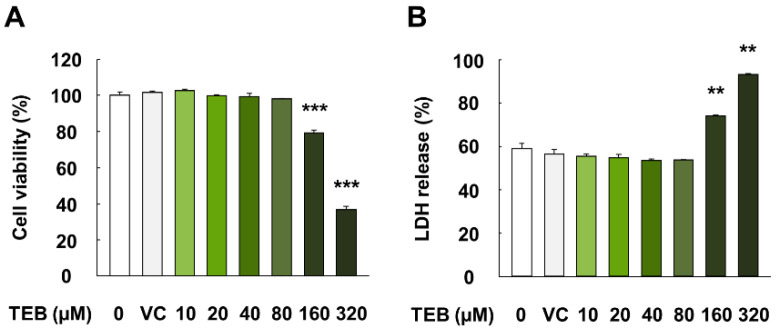
Cell viability and membrane integrity (LDH release) in HepG2 cells treated with TEB. (**A**) Percentage of cell viability and (**B**) LDH release in HepG2 cells. The cells were exposed to TEB (10, 20, 40, 80, 160, and 320 µM) or DMSO as a vehicle control (VC) for 24 h (*n* = 3 wells/group). The data are represented as mean ± SEM. ** (*p* < 0.01) and *** (*p* < 0.001) indicate a significant difference.

HepG2 cells exposed to TEB for 24 h did not show alterations in cell viability or LDH release at concentrations up to 80 µM (*p* > 0.05) (Figure 1A,B). However, cells exposed to 160 and 320 µM TEB for 24 h showed significantly lower viability and higher LDH release than the corresponding controls (*p* < 0.01). In addition, no significant difference was observed in cell viability and LDH release between the control (no treatment) and vehicle control (DMSO) (*p* > 0.05). Therefore, TEB concentrations of 20, 40, and 80 µM, which showed no cytotoxicity, were used for subsequent experiments.

### 3.2. Effects of TEB on Lipid Accumulation in HepG2 Cells

**Figure 2:**
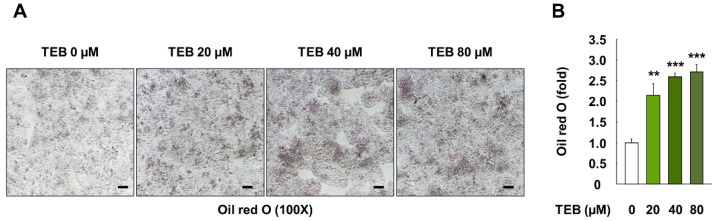
Lipid accumulation in HepG2 cells treated with TEB. (**A**) Microscopy images (100×, black bar = 100 µm) and (**B**) quantification of areas stained with Oil Red O in HepG2 cells. The cells were exposed to 20, 40, and 80 µM TEB for 24 h (*n* = 3 wells/group). The data are represented as mean ± SEM. ** (*p* < 0.01) and *** (*p* < 0.001) show a significant difference, compared to the control (DMSO).

HepG2 cells were stained with Oil Red O to evaluate whether TEB influenced lipid accumulation. Cells exposed to TEB (20, 40, and 80 µM, 24 h) showed prominent lipid accumulation, compared to the control (Figure 2A). Further, results from the quantification of the Oil Red O-stained areas showed markedly higher levels of lipid accumulation, compared to the control cells due to the influence of TEB (*p* < 0.01) (Figure 2B).

### 3.3. Effects of TEB on Lipid Uptake Regulation Proteins in HepG2 Cells

**Figure 3:**
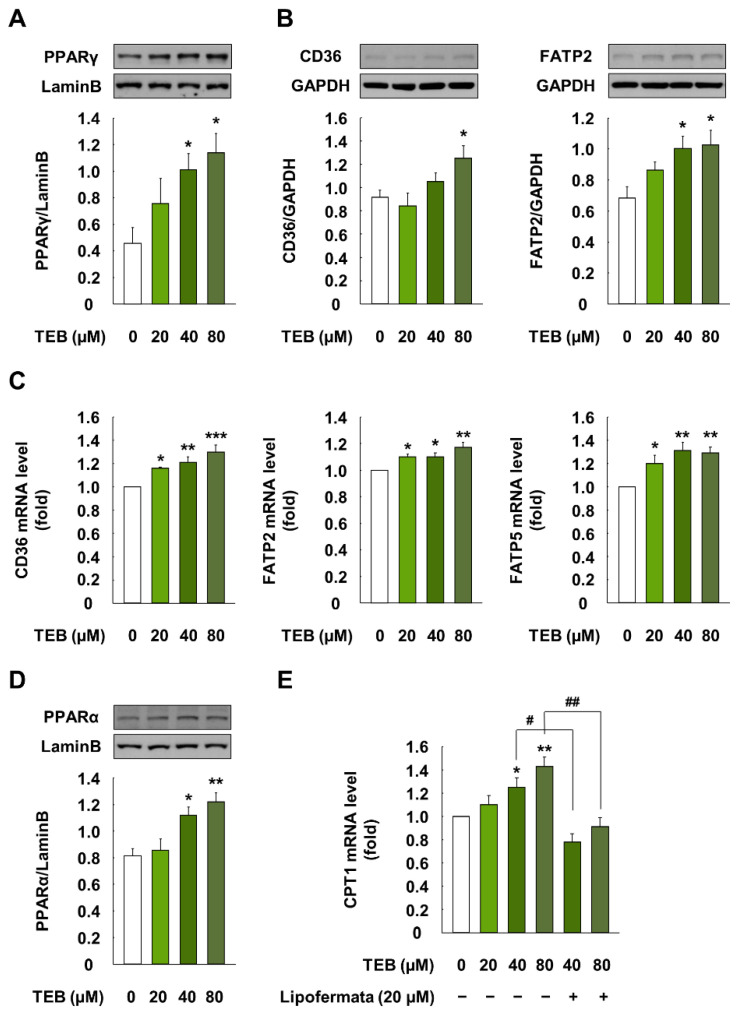
Expression of lipid uptake and lipid oxidation-associated molecules in HepG2 cells when treated with TEB. (**A**) Protein level of nuclear PPARγ (1:3,000 dilution) in HepG2 cells; (**B**) protein and (**C**) mRNA levels of CD36 (1:3,000 dilution), FATP2 (1:3,000 dilution), and FATP5 (1:3,000 dilution) in cells; (**D**) protein levels of PPARα (1:1000 dilution) in the nucleus and (**E**) mRNA level of CPT1 in cells. Cells were exposed to 20, 40, and 80 µM TEB for 1 or 12 h (*n* = 3 wells/group). Lipofermata (20 µM) was added to the cells 1 h before the TEB treatment. Lamin B and GAPDH were used as housekeeping protein and gene, respectively. The data are represented as mean ± SEM. * (*p* < 0.05), ** (*p* < 0.01), and *** (*p* < 0.001) show a significant difference, compared to the control (DMSO). # (*p* < 0.05) and ## (*p* < 0.01) show a significant difference, compared to the TEB-treated cells.

Treatment of cells with TEB (20, 40, and 80 µM for 1 h) significantly increased the translocation of PPARγ from the cytosol to the nucleus, compared with the control cells (*p* < 0.05) (Figure 3A). Further, cells treated with TEB (20, 40, and 80 µM, 12 h) showed higher protein levels of CD36 and FATP2 than the control cells (*p* < 0.05) (Figure 3B). TEB also markedly increased the gene levels of CD36, FATP2, and FATP5, compared to the control (*p* < 0.05) (Figure 3C).

### 3.4. Effects of TEB on Lipid Uptake Regulation in HepG2 Cells

Cells exposed to 40 and 80 µM TEB for 12 h showed significantly increased nuclear translocation of PPARα, compared to the control cells (*p* < 0.05) (Figure 3D). In addition, TEB-treated cells (40 and 80 µM, 12 h) had markedly higher mRNA levels of carnitine palmitoyltransferase (CPT) 1, compared to the control (*p* < 0.05) (Figure 3E). However, the pretreatment of cells with Lipofermata (20 µM for 1 h), a specific inhibitor of FATP2, downregulated the mRNA levels of CPT1, compared to those in the TEB-treated cells (*p* < 0.05).

### 3.5. Effects of TEB on Oxidative Stress in HepG2 Cells

**Figure 4:**
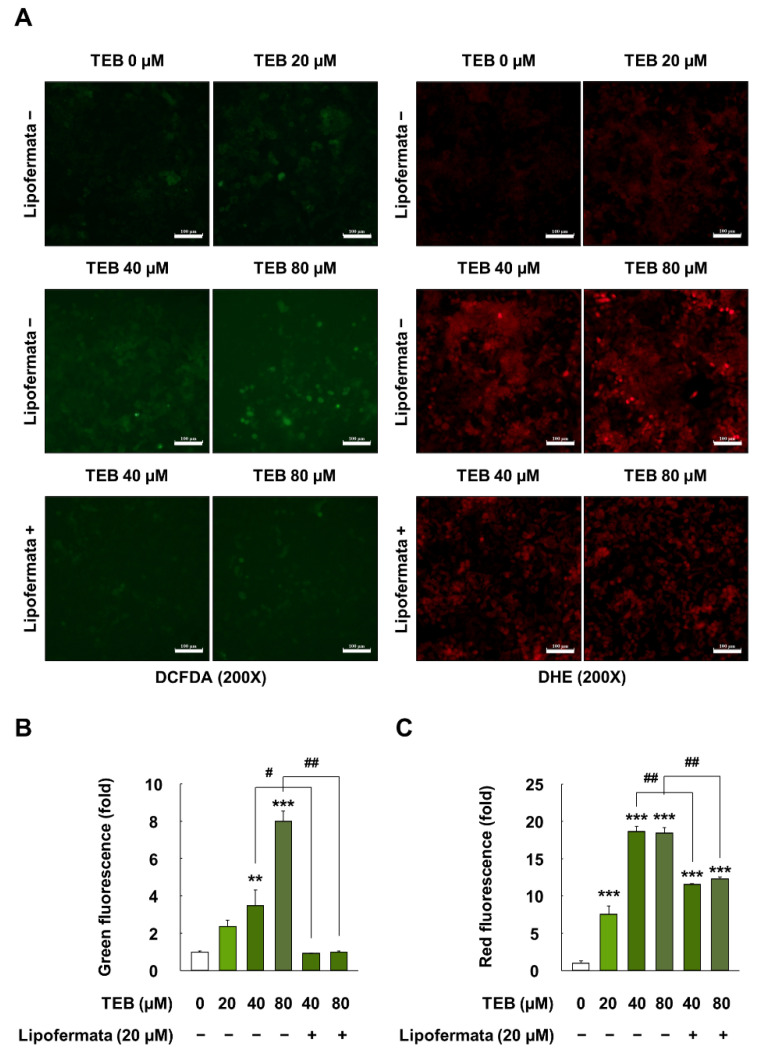
Oxidative stress measurements in HepG2 cells treated with TEB. (**A**) Fluorescence images of hydrogen peroxide (green) and superoxide (red) in cells (200× magnification, white bar = 100µm); quantification of the (**B**) green and (**C**) red fluorescence ratio in cells. Cells were exposed to 20, 40, and 80 µM TEB for 12 and 24 h with or without pretreatment with 20 µM Lipofermata for 1 h (*n* = 3 wells/group). The data are represented as mean ± SEM. ** (*p* < 0.01) and *** (*p* < 0.001) show a significant difference, compared to the control (DMSO). # (*p* < 0.05) and ## (*p* < 0.01) show a significant difference, compared to the TEB-treated cells.

HepG2 cells exposed to 20, 40, and 80 µM for 24 h showed 2.4-, 3.5-, and 8.0-fold higher levels of intracellular hydrogen peroxide than the control (*p* < 0.05) (Figure 4A,B). Intracellular superoxide levels in TEB (20, 40, and 80 µM for 24 h)-treated cells also increased by up to 7.6-, 18.6-, and 18.4-fold, respectively, when compared with the control (*p* < 0.05) (Figure 4A,C). However, the pretreatment of cells with Lipofermata (20 µM for 1 h) attenuated the oxidative stress (e.g., generation of hydrogen peroxide and superoxide) induced by TEB exposure (*p* < 0.05).

### 3.6. Effect of TEB on MMP and Lipid Export in HepG2 Cells

**Figure 5:**
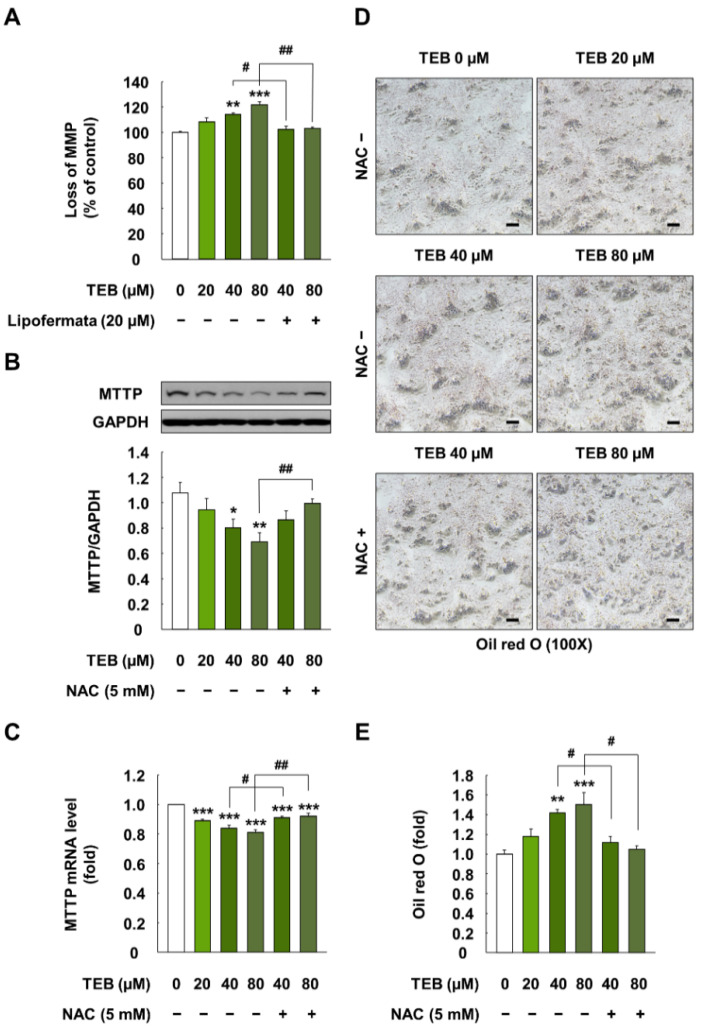
MMP and expression of lipid export-associated cellular indicators in HepG2 cells treated with TEB. (**A**) Loss of MMP in HepG2 cells; (**B**) protein and (**C**) gene expression levels of MTTP (1:3000 dilution) in HepG2 cells; (**D**) microscopy images (100×, black bar = 100 µm) and (**E**) quantification of areas stained with Oil Red O in cells. The cells were exposed to 20, 40, and 80 µM TEB for 24 h with or without pretreatment with 20 µM Lipofermata or 5 mM NAC for 1 h (*n* = 3 wells/group). GAPDH was used as a housekeeping control. The data are presented as mean ± SEM. * (*p* < 0.05), ** (*p* < 0.01), and *** (*p* < 0.001) show a significant difference, compared to the control (DMSO). # (*p* < 0.05) and ## (*p* < 0.01) show a significant difference, compared to the TEB-treated cells.

The treatment of cells with TEB (40 and 80 µM for 24 h) markedly increased the loss of MMP, compared to that in the control cells (*p* < 0.05) (Figure 5A). However, the pretreatment of cells with Lipofermata (20 µM for 1 h) attenuated the loss of MMP, compared to that in the corresponding TEB-exposed cells (*p* < 0.05).

TEB-treated cells (20, 40, and 80 µM for 24 h) showed decreased protein and mRNA levels of MTTP, compared to the control (*p* < 0.05) (Figure 5B,C). However, the pretreatment of cells with NAC (5 mM, 1 h) restored the MTTP levels to those in the control (*p* < 0.05) (Figure 5B,C). Moreover, TEB (20, 40, and 80 µM, 24 h)-induced lipid accumulation was significantly decreased when the cells were treated with NAC (5 mM, 1 h) (*p* < 0.05) (Figure 5D,E).

## 4. Discussion

Despite increasing concerns about pesticide residues in food products and water, various pesticides have been widely used to control plant diseases and improve crop yield. A previous study showed that insecticide exposure impaired lipid metabolism, thereby resulting in the development of NAFLD [21]. Moreover, recent studies have reported that fungicides such as propiconazole, flutriafol, cyproconazole, dazomet, fluazinam, hexaconazole, pyrasulfotole metabolite, and myclobutanil can cause NAFLD pathogenesis [22,23,24,25]. However, the toxic effects of TEB on lipid accumulation and lipid metabolism in the liver have rarely been studied. Therefore, we evaluated the risk of TEB exposure in the development and progression of NAFLD, particularly lipid accumulation and associated cellular molecular mechanisms, using the human liver cell line, HepG2.

NAFLD development is initiated by the accumulation of lipids in the liver due to the imbalance between lipid uptake and export. Therefore, we first evaluated lipid accumulation at concentrations of up to 80 µM TEB, which did not affect the cell viability and membrane integrity of HepG2 cells. Our results indicated that TEB-treated cells had significantly higher lipid accumulation. Furthermore, TEB-treated cells showed increased protein and gene expression levels of CD36 and FATP with the nuclear translocation of PPARγ. According to a previous study, TEB induced triglyceride accumulation in HepaRG cells through the pregnane X receptor [17,26]. The pregnane X receptor plays a pivotal role in xenobiotic metabolism by regulating CYP expression [17,26]. However, in addition to the pregnane X receptor, PPAR is also a transcriptional regulator of lipid metabolism; in particular, PPARγ has been reported to control lipid uptake-related proteins, such as CD36 and FATP [27]. In previous studies, mice on a high-fat diet showed liver steatosis through increased protein and gene levels of CD36, whereas the knockout of CD36 resulted in decreased hepatic lipid levels [28]. Moreover, the knockdown of FATP2 and FATP5 among the six FATP isoforms in the mammalian liver resulted in decreased fatty acid uptake in mice [29,30,31]. Therefore, our data indicate that TEB led to lipid accumulation in liver cells through an increase in lipid uptake-related proteins and genes such as CD36 and FATP.

Fatty acids that enter the cytoplasm through facilitation by CD36 and FATP undergo oxidation to generate energy [32]. Fatty acids are metabolized to fatty acyl-CoA and transferred to the mitochondria using CPT1 [33]. In this process, CPT1, a major controller of mitochondrial β-oxidation, regulates the entry of fatty acids via the conversion of acyl-CoA to acyl carnitine [34], and the transcription of CPT1 is controlled through the activation of PPARα [35]. In our study, TEB increased the nuclear translocation of PPARα and the mRNA level of CPT1 in HepG2 cells. However, the pretreatment of cells with Lipofermata, a pharmacological inhibitor of FATP2, attenuated the mRNA level of CPT1. In a previous study, fatty acids accumulation in the liver was found to induce mitochondrial β-oxidation [36], and an increase in CPT1 expression through PPARα activation was observed in the livers of rodents and patients with NAFLD [37]. These previous studies along with our data indicate that fatty acid accumulation in the liver increases mitochondrial β-oxidation through PPARα and CPT1. However, the increase in mitochondrial β-oxidation is known to overproduce considerable amounts of ROS and oxidative stress [38]. In our study, TEB significantly increased oxidative stress (i.e., hydrogen peroxide and superoxide) and MMP loss in HepG2 cells, whereas a decrease in fatty acid accumulation by Lipofermata attenuated the generation of oxidative stress and MMP loss. Indeed, previous clinical studies have demonstrated that fatty acid β-oxidation in the mitochondria of the liver was initially increased in response to lipid accumulation [39,40]. However, the inability to control excessive hepatic fatty acid accumulation resulted in ROS generation through uncoupled oxidative phosphorylation in the mitochondria [41]. In addition, this sustained ROS generation progressively impaired respiratory chain activity, resulting in complete mitochondrial dysfunction [42]. Therefore, our data revealed that TEB can induce oxidative stress and mitochondrial dysfunction (i.e., MMP loss) by increasing mitochondrial β-oxidation via the activation of PPARα and CPT1.

Lipid export is the only means for the decrease of liver lipid content in addition to mitochondrial β-oxidation [43]. However, fatty acids can only be secreted from the liver in the form of VLDL because of their hydrophobic nature [44]. Therefore, apolipoprotein B 100 and MTTP, which are related in hepatic VLDL assembly and secretion, are known as major factors in the export of triglycerides [45]. In particular, MTTP has been reported to regulate lipoprotein assembly through the transfer of lipids to apolipoprotein B 100 and the ionic interaction between MTTP and the N-terminus of apolipoprotein B 100 [46]. In our study, TEB-treated cells showed lower MTTP protein and mRNA levels, compared to the control cells. This indicates that TEB downregulated lipid export. However, the inhibition of oxidative stress using NAC recovered the protein and mRNA levels of decreased MTTP. Similar to our data, a previous study reported that MTTP-deficient patients showed impaired exports of VLDL from the liver, thereby resulting in the development of steatosis with the accumulation of triglycerides in the liver [47]. Moreover, a recent study indicated that excessive lipid overload-induced oxidative stress resulted in the suppression of MTTP through protein kinase C delta and hepatocyte nuclear factor 4 alpha, which impaired VLDL secretion in the liver of fish [48]. Similar to these data, our results showed that the suppression of oxidative stress using the antioxidant NAC caused a lower level of lipid accumulation, compared to that in TEB-treated cells. Collectively, our data revealed that oxidative stress induced by excessive mitochondrial β-oxidation in TEB-treated cells resulted in impaired lipid export through a decrease in MTTP expression.

Collectively, our data demonstrated that TEB exposure can induce lipid accumulation in HepG2 cells by increasing lipid uptake, generating oxidative stress via excessive activation of mitochondrial β-oxidation, and impairing lipid export. Cellular oxidative stress through mitochondrial β-oxidation was identified as a major pathway in the disruption of lipid metabolism. Although further in vivo studies are required to elucidate the effect of TEB on lipid accumulation and lipid metabolism, our data suggest that TEB exposure in humans can be a risk factor for NAFLD development and progression.

## Figures and Tables

**Table 1:** Primers used for RT-PCR analysis.

Gene ^1^	Primer Sequence 5′‒3′
CD36 (Human)	(F) AAC GGC TGC AGG TCA ACC TAT T (R) GGT CCC AGT CTC ATT AAG CCA AAG
FATP2 (Human)	(F) ATG GAG TGC ATG TGC CAG ATC A (R) TAG AAA CCG GGG CCT TGC ATA A
FATP5 (Human)	(F) GAA GGC AAC ATG GGC TTA GTC AAC (R) TGT CGA ACT GCA CCA GCT CAA A
CPT1 (Human)	(F) TTG CAG GCG AGA ACA CGA TCT T (R) CTG TAG GCC TTG GGA ACT TGG AAA
MTTP (Human)	(F) CAG TGC AGT TTT CCC AGT ACC CAT (R) CCT GTG GAC AGC CTT TCG TAC TTT
GAPDH (Human)	(F) GAC CCC TTC ATT GAC CTC AAC TAC (R) ATG ACA AGC TTC CCG TTC TCA G

^1^ CD, cluster of differentiation; FATP, fatty acid transporter protein; CPT1, carnitine palmitoyltransferase 1; MTTP, microsomal triglyceride transfer protein; GAPDH, glyceraldehyde 3-phosphate dehydrogenase.

## Data Availability

Not applicable.
